# Elaborated Action of the Human Primosome

**DOI:** 10.3390/genes8020062

**Published:** 2017-02-08

**Authors:** Andrey G. Baranovskiy, Tahir H. Tahirov

**Affiliations:** Eppley Institute for Research in Cancer and Allied Diseases, Fred & Pamela Buffett Cancer Center, University of Nebraska Medical Center, Omaha, NE 68198, USA; abaranovskiy@unmc.edu

**Keywords:** DNA replication, human, primosome, primase, DNA polymerase α, protein-DNA interaction, RNA synthesis, initiation, termination, steric hindrance

## Abstract

The human primosome is a 340-kilodalton complex of primase (DNA-dependent RNA polymerase) and DNA polymerase α, which initiates genome replication by synthesizing chimeric RNA-DNA primers for DNA polymerases δ and ε. Accumulated biochemical and structural data reveal the complex mechanism of concerted primer synthesis by two catalytic centers. First, primase generates an RNA primer through three steps: initiation, consisting of dinucleotide synthesis from two nucleotide triphosphates; elongation, resulting in dinucleotide extension; and termination, owing to primase inhibition by a mature 9-mer primer. Then Polα, which works equally well on DNA:RNA and DNA:DNA double helices, intramolecularly catches the template primed by a 9-mer RNA and extends the primer with dNTPs. All primosome transactions are highly coordinated by autoregulation through the alternating activation/inhibition of the catalytic centers. This coordination is mediated by the small C-terminal domain of the primase accessory subunit, which forms a tight complex with the template:primer, shuttles between the primase and DNA polymerase active sites, and determines their access to the substrate.

## 1. Introduction

In all eukaryotic organisms, genome replication depends on activity of the primosome, a four-subunit complex of DNA primase and DNA polymerase α (Polα) [1]. The primosome initiates synthesis of both the leading and lagging strands by making chimeric RNA-DNA primers, which are required for the loading of replication factor C (RFC), proliferating cell nuclear antigen (PCNA), and replicative DNA polymerases δ and ε [2,3]. At each origin, the primosome is involved only once for leading strand initiation, while it starts every Okazaki fragment on the discontinuously synthesized lagging strand. Given the sizes of Okazaki fragments (165-bp) and chimeric primers (30–35 nucleotides), the primosome synthesizes up to 20% of the lagging strand and, therefore, approximately 10% of the genome [4,5]. During maturation of the Okazaki fragments, both the RNA and a significant portion of the DNA track of a chimeric primer are being deleted [6]. As a result, DNA synthesized by Polα comprises approximately 1.5% of the mature genome [7]. These regions are mutation hotspots because Polα has relatively low fidelity due to the absence of proofreading activity. Thus, despite low retention of Polα-synthesized DNA tracks in the mature genome, the primosome has a large impact on genome stability and evolution. Recently, it has been shown that the primosome is responsible for generation of RNA-DNA fragments in the cytosol and that it regulates the activation of type I interferons [8].

The primosome synthesizes chimeric primers in a highly coordinated fashion. RNA primer synthesis by primase involves three steps: initiation, elongation, and termination [9,10]. During the initiation step, primase binds the DNA template and two cognate rNTPs (one at the initiation site and the other at the elongation [catalytic] site) and catalyzes the formation of a dinucleotide [11,12]. Extension of the RNA is restricted due to the intrinsic property of primase to terminate synthesis at a strictly defined point [13]. Then Polα intramolecularly captures the mature RNA primer for subsequent extension by dNTPs [11,14,15]. Recent breakthroughs in structural studies of the human primosome [13] and its components [16,17,18,19,20,21,22] (Table 1) allow for accurate modeling of the primosome conformations during all stages of chimeric primer synthesis.

## 2. Organization of the Human Primosome

**Figure 1:**
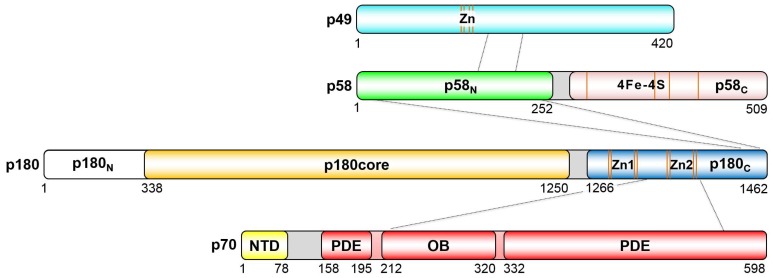
Schematic representation of the domain organization in the human primosome. The borders of the regions participating in intersubunit interactions are designated by dotted lines. Positions of the conserved cysteines coordinating zinc or [4Fe-4S] cluster are indicated by orange lines. The linkers responsible for flexible connections between domains are colored gray.

Human Polα belongs to the B family of DNA Pols and is comprised of a 166-kDa catalytic subunit (p180) and a 66-kDa accessory subunit (p70). The catalytic domain of p180 (p180core) possesses DNA-polymerizing activity but has no proofreading exonuclease activity, in contrast to other replicative DNA Pols, δ and ε. The C-terminal domain of p180 (p180_C_) is flexibly connected to a catalytic core by a 15-residue-long linker, and it contains two conserved zinc-binding modules, Zn1 and Zn2 (Figure 1), where each zinc is coordinated by four cysteines [19,24]. Zn2 and the helical region between the two zinc-binding modules provide the extended interaction interface (~4000 Å^2^) with p70, while the short peptide (1447–1455) mediates the interaction between Polα and primase [13,19]. The N-terminus of p180 is predicted to be poorly folded and has no conserved motifs required for primosome function. The structural information for this region is limited to a small peptide in the catalytic subunit of yeast Polα (residues 140–147) that mediates interaction with the replisome [25]. The accessory B subunit (p70; also known as p68) consists of a globular N-terminal domain (NTD or p70_N_), a catalytically dead phosphodiesterase domain (PDE), and an oligonucleotide/oligosaccharide-binding (OB) domain. The OB domain is embedded into the PDE domain, representing the common feature of B-family DNA Pols [19,26]. The globular NTD is attached to the PDE via a long flexible linker and participates in interactions with other DNA replication proteins [19,27,28].

Human primase consists of a 50-kda catalytic subunit (p49; also known as p48, PRIM1, Pri1, and PriS) and a 59-kDa regulatory subunit (p58; also known as PRIM2, Pri2, and PriL) (Figure 1). Eukaryotic and archaeal primases have a similar structural organization, which indicates a common evolutionary ancestor [29]. In contrast to prokaryotic primases, the zinc-binding motif of eukaryotic/archaeal primases is integrated into the “prim” fold of the catalytic subunit and probably plays only a structural role [16,18,30,31,32]. p58 has two distinct domains: the N-terminal domain (p58_N_) with a mixed α/β-fold and the all-helical C-terminal domain (p58_C_), connected by an 18-residue linker (253–270) [18]. Similar to yeast primase [33], four cysteines of p58_C_ coordinate an iron-sulfur cluster ([4Fe-4S]) which is buried inside of the domain and is important for p58_C_ folding [21,22,34,35].

There was one report claiming that all four *Saccharomyces cerevisiae* B-family DNA polymerases coordinate the [4Fe-4S] cluster at the second cysteine-rich module (referred to here as Zn2) of the C-terminal domain of the catalytic subunits (CTD, analog of p180_C_) [36]. However, the provided experimental evidence was uncertain for Polα. For example, Polα CTD purified under anaerobic conditions contained only 0.1 mol non-heme iron and acid-labile sulfide per mol CTD, while CTDs of other B-family DNA polymerases (δ, ε, and ζ) contained 2.0 to 2.6 Fe and S per monomer. Coordination of the [4Fe-4S] cluster by Polα CTD has not been confirmed in subsequent studies where high-purity stoichiometric Polα complexes have been obtained [37,38]. Structural studies of yeast and human Polα do not support the presence of an iron-sulfur cluster in Polα CTD; only two zinc ions coordinated by Zn1 and Zn2 modules were seen [13,19,39]. Zn2 is important for interaction between Polα subunits and snugly fits the docking site on the OB domain. Coordination of an [4Fe-4S] cluster by the Zn2 module would certainly change its shape and disrupt the interaction between the catalytic and B subunits. It was also shown that partially purified Polε CTD contained significant levels of iron, whereas its complex with the B subunit was iron-free [37]. These data support the idea that the CTDs of Polα and Polε with an inadvertently misincorporated iron-sulfur cluster cannot form stable complexes with the corresponding B subunits. It is worth noting, that placing an affinity tag on the B subunit is crucial for obtaining stoichiometric complexes of B-family DNA polymerases, because it prevents the contamination of preparations with a free catalytic subunit.

**Figure 2:**
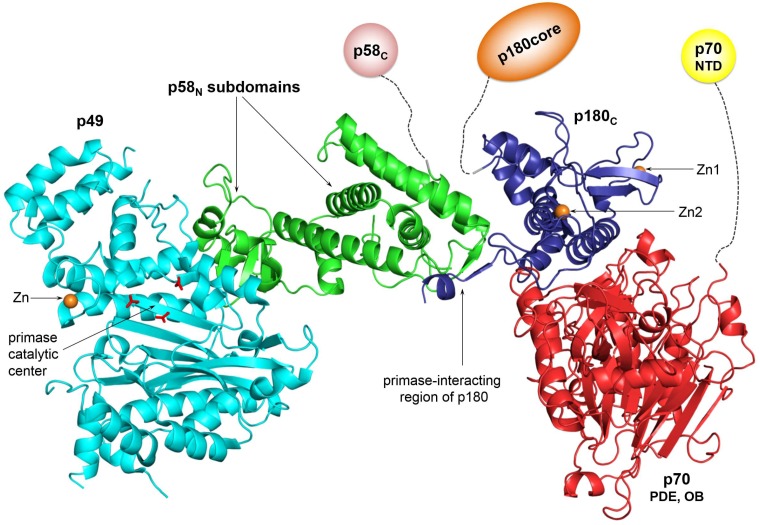
The platform of the human primosome. Coordinates of the human primosome (PDB ID 5EXR) were used to represent the platform structure. The color scheme for domains is the same as in Figure 1. The positions of p58_C_ and p180core, as well as the linkers connecting them to the platform, vary depending on the primer synthesis step. For space-saving purposes, p58_C_, p180core, and p70-NTD are shown at reduced scale relative to the platform. All figures were prepared using the PyMOL Molecular Graphics System (version 1.8, Schrödinger, LLC).

Substantial conformational changes in the primosome are essential for seamlessly carrying out the entire cycle of RNA-DNA primer synthesis. The primosome has three functional centers: the RNA- and DNA-polymerizing centers, located on p49 and p180core, respectively [11,40,41], and regulatory p58_C_, which is responsible for template:primer binding and translocation from primase to Polα [42,43]. The structure of the human primosome reveals an elongated platform p49-p58_N_-p180_C_-p70 (Figure 2) that can hold p180core and p58_C_ either stationary, by docking in inactive form, or flexibly, by linkers during various stages of primer synthesis [13]. Interestingly, the points of the linker’s attachment to the platform are fairly close despite their origination from different subunits. The platform itself has limited flexibility because p58_N_ subdomains were shown to oscillate by several degrees relative each other [13,16,18]. p58_N_ could be considered as a core of the platform; its smaller subdomain interacts with p49, while the larger, α-helical subdomain interacts with p180_C_ and is connected by the linker to p58_C_ (Figure 2). Such organization of the primosome provides significant freedom for the functional centers in their movement relative to each other.

## 3. Interaction of Human Primase with a Template:Primer

**Figure 3:**
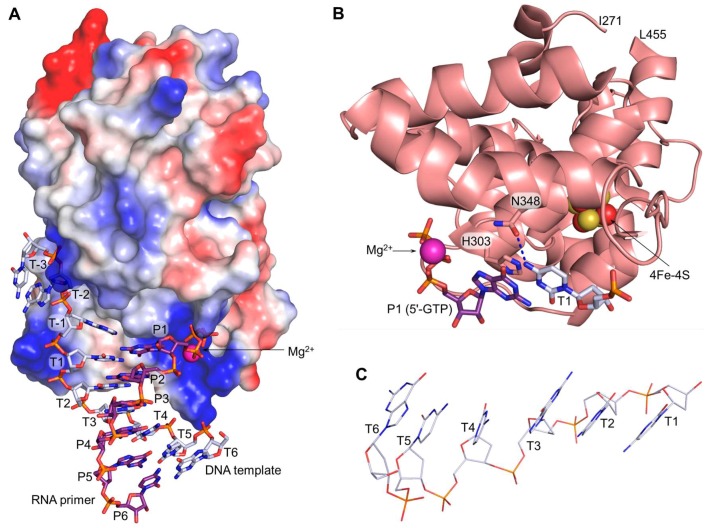
Interaction of p58_C_ with a DNA template primed by RNA. (**A**) p58_C_ specifically recognizes the DNA:RNA junction at the primer 5′-end containing the triphosphate. The p58_C_ surface is represented by the vacuum electrostatic potential at 20% transparency; (**B**) mechanism of p58_C_ specificity to a purine at the initiation site. The hydrogen bond is depicted by dashed blue line; (**C**) DNA template bends between T3 and T4. All parts of the figure were drawn using the coordinates of the p58_C_/DNA:RNA complex (PDB ID 5F0Q).

Recent biochemical and structural studies finally unveiled the mechanism of human primase interaction with a DNA template and an RNA primer, where p58_C_ firmly holds the DNA:RNA duplex while p49 catalyzes the attachment of rNTPs to the 3′-end of the primer [13,43]. p58_C_ specifically recognizes the junction at the 5′-end of the RNA primer, which contains the 5′-triphosphate group (Figure 3A). The β- and γ-phosphates of the triphosphate moiety make six hydrogen bonds with p58_C_, explaining the critical role of these phosphates in primase activity and their affinity for the DNA:RNA substrate [43,44,45]. Moreover, recognition of the 5′-triphosphate prevents p58_C_ rotation around the duplex, thereby strictly determining the position and orientation of p58_C_ relative to the platform and p180core during all primosome transactions. Coordination of a divalent metal probably stabilizes the conformation of the triphosphate group and its complex with p58_C_. Arg-306 interacts with both the β- and γ-phosphates and is critical for primase activity, especially during dinucleotide synthesis [16,42]. There are no other contacts between p58_C_ and the RNA primer except for stacking between His-303 and the base of the 5′-GTP (Figure 3B).

The structure of p58_C_/DNA:RNA revealed the location and organization of the initiation site with bound initiating GTP which forms the 5′-end of the nascent dinucleotide [13]. The critical role of p58_C_ in binding the initiating nucleotide explains why p49 is able to extend RNA fragments but cannot initiate synthesis from two rNTPs [11]. The relatively weak coordination of the initiating rNTP by only six hydrogen bonds explains the low affinity of this site (*K_m_*(ATP) = 3 mM), which is 11-fold lower compared to the elongation site [11]. Human primase has no obvious sequence specificity except the well-known preference of the initiation site for GTP/ATP [9,46], which is probably due to the cumulative effect of two factors. First, His-303 demonstrates good stacking with the initiating purine, while its ring would only partially overlap with a pyrimidine base (Figure 2B). Secondly, Asn-348 can use its carbonyl or amino group to form a hydrogen bond with N4 or O4 of the templating cytidine or thymine, respectively.

p58_C_ forms 13 hydrogen bonds with the template, the majority of which are located near the junction. The presence of 19 hydrogen bonds between p58_C_ and DNA:RNA results in a stable complex with a *K_d_* of 32.7 nM [43]. For comparison, the catalytic core domains of human Polε and Polα bind the template:primer with 2.4-fold and 10-fold lower affinity, respectively [20,47]. The intact human primase and p58_C_ have similar affinities for DNA:RNA, supporting the idea that p58_C_ is a major DNA-binding domain in the primosome [43]. The primer 5′-triphosphate and the template 3′-overhang exhibit a synergistic effect on duplex binding by primase and its RNA-polymerizing activity [43]. The dependence of p58_C_ affinity on the stability of the DNA:RNA duplex explains the abortive character of RNA synthesis at the beginning of the elongation stage and in the case of AT-rich templates [12,18]. The structure of p58_C_/DNA:RNA complex explains why the His-401-Arg mutation in yeast primase leads to lethality [34]. The bulky side chain of the arginine in place of His-351 (corresponds to His-401 in yeast primase) disrupts the interaction with DNA:RNA because of steric hindrance with the template and/or the DNA-interacting loop containing residues 355–366. p58_C_ affects the template conformation in the DNA:RNA duplex: it maintains the B-DNA conformation of the template deoxyriboses that are in contact with p58_C_ (T1–T3), while three nucleotides at the 5′-end (T4–T5) are in the A-DNA conformation (Figure 3C).

## 4. Mechanisms of RNA Synthesis Initiation, Elongation, and Termination

**Figure 4:**
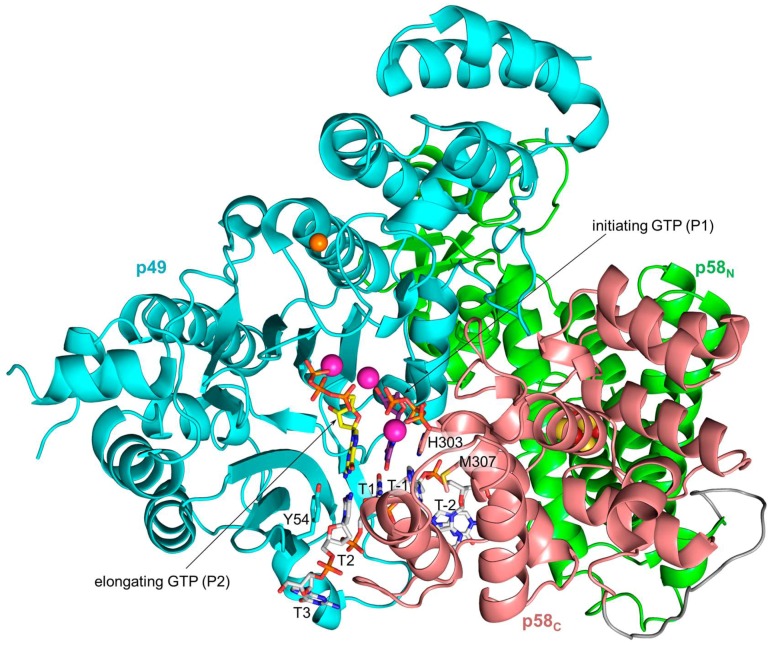
The model of human primase in the initiation complex with a DNA template and two GTP molecules. The linker between p58_N_ and p58_C_ colored gray is shown for reference purposes only. The carbons of the DNA template, initiating GTP, and elongating GTP are colored gray, purple, and yellow, respectively. The atoms of zinc, magnesium, iron, and sulfur are represented as spheres and colored orange, magenta, red, and yellow, respectively.

**Figure 5:**
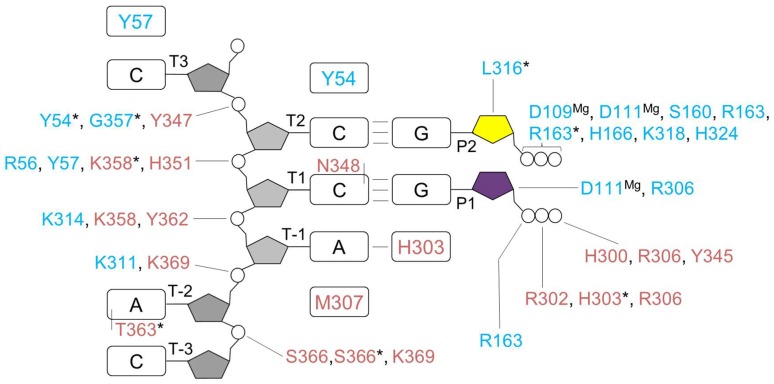
Interaction of human primase with a DNA template and rNTPs during RNA synthesis initiation. The color scheme is the same as in Figure 4. The residues of p49 interacting with the DNA template and the initiating GTP are identified from the model of the initiation complex. The asterisk indicates that a main-chain atom of the amino acid forms a hydrogen bond with a nucleotide. Amino acids participating in stacking interactions with nucleotides are shown in rectangular boxes. Interactions of aspartates 109 and 111 with both rNTPs are mediated by the Mg^2+^ ions.

The structure of p58_C_/DNA:RNA (PDB ID 5F0Q) together with the structures of p49–p58 (PDB ID 4RR2) [18] and p49–p58(1–253)/UTP (PDB ID 4BPW) [16] allows for obtaining accurate models of primase during all steps of RNA synthesis [13]. Structure-based modeling by superimposition of the second nucleotide of the primer from the p58_C_/DNA:RNA complex with UTP bound at the elongation site of p49 reveals the compact initiation complex (Figure 4) with good shape complementarity and eight potential hydrogen bonds between p49 and p58_C_ [13]. This organization of the initiation complex where the active site is shared by p49 and p58_C_ results in cooperative binding of four template nucleotides and initiating rNTP (Figure 5). The active site is able to accommodate only three template nucleotides which are placed between Tyr-54 of p49 at the 5′-end and Met-307 of p58_C_ at the 3′-end. p49 can make only six hydrogen bonds with a template because of its shallow DNA-binding interface (Figure 5). The active site elements accommodated by two flanking β-sheets of p49 are adopted for the common mechanism of nucleic acids synthesis through the coordination of two divalent metals [48].

The model of the initiation complex revealed that p49 participates in pre-catalytic positioning of the initiating GTP by making three hydrogen bonds: Arg-163 with the α-phosphate, Asp-306 with the O2′ of a ribose, and the bond between Asp-111-coordinated Mg^2+^ and the O3′ of a ribose (Figure 5). During the elongation stage of RNA synthesis, the initiation site disintegrates due to the growing distance between its structural elements provided by both subunits: p58_C_ continues holding the 5′-end of the primer, while p49 is establishing the above-described three hydrogen bonds with the growing 3′-end, because during primer extension the 3′-terminal nucleotide occupies the same space on p49 as the initiating rNTP. The interaction between the O2′ of the initiating GTP and Asp-306 of p49 explains the strict preference for ribonucleotides at the initiation step [46]. Consistently, the primase is also sensitive to the presence of the O2′ at the primer 3′-terminus during its extension [38,49]. Replacement of Asp-306 by Ala severely affects primase activity, but to a lesser extent compared to alanine substitutions of Asp-109 or Asp-111 which coordinate the catalytic Mg^2+^ ions [41]. In contrast, the elongation site demonstrates low selectivity for rNTPs [38,49], compensated for by a 10- to 130-fold higher cellular concentration of rNTPs versus dNTPs [50]. Therefore, the probability of dNTP insertion, which works as a chain terminator for primase, is a rare event in vivo. Selectivity of the initiation site to ribose, mediated by the hydrogen bond between Asp-306 and the O2′, is probably due to the requirement for accurate positioning of the O3′, which is deprotonated by Mg^2+^ for the nucleophilic attack on the α-phosphate of the incoming NTP. Moreover, such selectivity potentially prevents the primase from extending DNA tracks made by Polα or other DNA Pols. It is quite possible that primase binds all three substrates before formation of the initiation complex, which works as a locking mechanism and fixes the substrates in catalytically proficient position.

**Figure 6:**
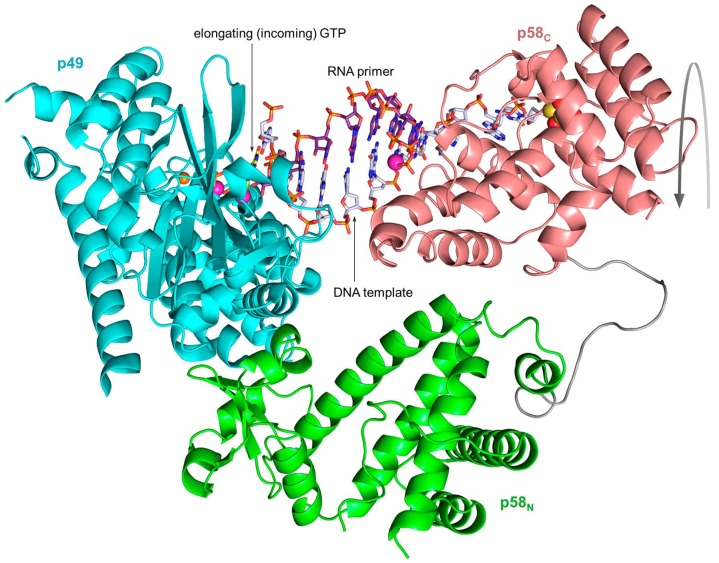
The model of human primase in elongation complex with a DNA template, primed by 7-mer RNA, and an incoming GTP. The curved arrow shows the direction of p58_C_ rotation relative to p49-p58_N_ during primer extension. The atoms of zinc, magnesium, iron, and sulfur are represented as spheres and colored orange, magenta, red, and yellow, respectively.

Modeling [13,18] and mutational [16] studies indicate that p49 employs the same amino acids for interactions with the DNA template during the initiation and elongation steps of primer synthesis. The weak interaction between p49 and the template-primer [43] suggests the mechanism of primase translocation along the template: p49 dissociates from DNA:RNA, held by p58_C_, after each round of nucleotide incorporation and quickly rebinds it by placing the 3′-terminal nucleotide of the primer at the binding site for the initiating nucleotide or, more exactly, to its section located on the catalytic subunit. In accordance with biochemical data [43], the model of the elongation complex (Figure 6) revealed a lack of interaction between human primase and the emerging RNA strand, except for the same contacts as found in the initiation complex (Figure 5). The open architecture of the primase/DNA:RNA complex, where contacts with both the minor and major grooves are absent, explains the ability of DNA primases to extend mispaired primer termini and perform translesion synthesis [51,52].

Due to the tight association with the template:primer junction [43], p58_C_ must move away from p49 during primer extension, by following the helical path of the growing DNA:RNA duplex [13]. Probably, such spiral movement of p58_C_ defines the mechanism of the primase counting phenomenon, which results in primer synthesis termination [12,18,43,53]. The model of the elongation complex, where primase is ready to generate an 8-mer primer, demonstrates that p58_C_ is in proximity to the helical subdomain of p58_N_ (Figure 6). Extension of the 8-mer primer would be complicated because of the emerging steric hindrance between the two p58 domains, which compromises the pre-catalytic alignment of the O3′ of a primer and the α-phosphate of an incoming NTP. The plasticity of p58_N_ allows primase to overcome steric hindrance during synthesis of the 9-mer primer but not during the following extension step [13]. Due to this plasticity, the intra-subunit steric hindrance works as a molecular brake to stop primase, which results in an RNA primer with a well-defined length optimal for utilization by Polα. The linker between p58_N_ and p58_C_ is not important for RNA synthesis termination because its shortening did not reduce the size of RNA products [18]. In contrast, primase pausing is dependent on the strength of the p58_C_/DNA:RNA complex; that is why its disturbance by changes in p58_C_ sequence [42] and the template:primer structure [43] attenuates the counting effect.

Salt, the type of divalent metal, and the metal’s concentration affect the distribution of RNA synthesis products [43]. Moreover, the *de novo* assay masks the effect of synthesis termination on templates, forming stable duplexes with 9-mer RNA primers, due to a 6000-fold lower primase affinity for single-stranded DNA versus a primed one [18,43]. On the other hand, 9-bp AT-rich DNA-RNA duplexes are not stable at common reaction conditions (30–35 °C), which significantly reduces the probability of RNA synthesis restart. Modeling of elongation complexes with 9 to 11-mer primers indicates that the steric hindrance is predominant only upon synthesis of 10- and 11-mer RNA [13]. If Polα is absent in the reaction, primase occasionally bypasses this barrier, using DNA:RNA substrates dissociated from p58_C_, which results in the accumulation of longer products upon extended incubation.

## 5. Mechanism of RNA Primer Transfer to Polα and Its Extension with dNTPs

**Figure 7:**
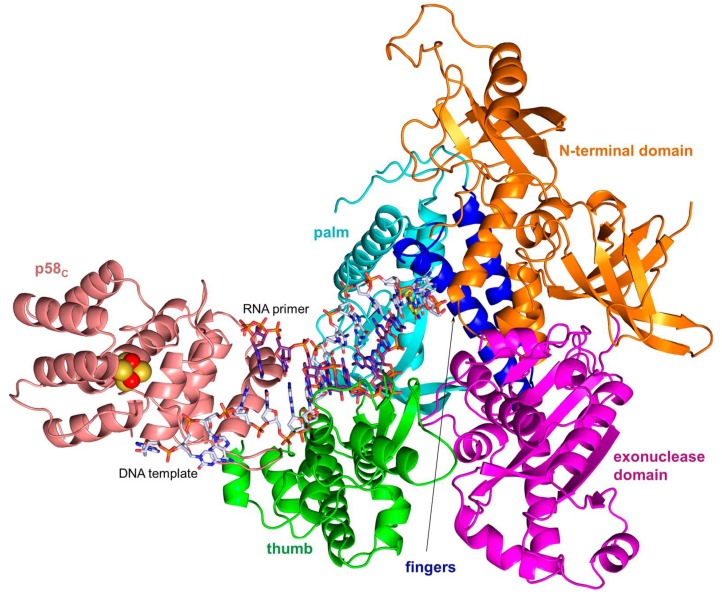
The model of the switch complex containing p180core, p58_C_, a DNA template primed by a 9-mer RNA, and incoming dCTP. p180core subdomains are shown in different colors. The carbons of the DNA template, RNA primer, and incoming dCTP are colored gray, purple, and yellow, respectively. This model was made using the coordinates of the p180core/DNA:RNA/dCTP complex (PDB ID 4QCL) and p58_C_/DNA:RNA complex (PDB ID 5F0Q).

According to biochemical data, upon completion of RNA primer synthesis p58_C_ continues to hold the template-primer until Polα captures it [11,12]. Recent structural data support this observation by showing that the predominant length of RNA primers is nine nucleotides and the optimal substrate for Polα is a 9-bp DNA:RNA duplex [17,43]. These data indicate that p58_C_ and p180core will form a switch complex before Polα starts an extension of the RNA primer with dNTPs. The model of this complex revealed the concurrent binding of a 9-bp DNA:RNA duplex and shape complementarity between both subunits (Figure 7). According to this model, p58_C_ will not allow Polα to extend shorter duplexes because the 3′-end of the primer does not reach the active site. Finally, biochemical experiments confirmed the idea that Polα in the primosome extends only the mature 9-mer RNA primers [13].

Similar to other B-family DNA polymerases and their prototypes from viruses, bacteriophages and bacteria, p180core has a “right-hand” fold: an active site formed by a “palm” holding the catalytic residues and making a set of interactions with three base pairs of the DNA double helix at the 3’-end of a primer, a “thumb” that secures the polymerase grip onto the template-primer helix, and “fingers” providing the induced-fit closure of the active site after binding of the cognate dNTP (Figure 7). Polα cannot correct its own mistakes during DNA copying because of evolutionary substitution of the catalytic amino acid residues in the exonuclease active site [54].

Polα possesses an interesting feature of binding and extending DNA:RNA and DNA:DNA duplexes with similar efficiency [20,38,55]. Structural data for p180core in ternary complex with DNA:RNA/dCTP and in binary complex with DNA:DNA indicate that Polα binds the DNA and hybrid duplexes in a similar way [17,20]. There are no significant conformational changes in p180core to accommodate different duplexes; instead, Polα imposes the A-DNA conformation on the DNA primer [20] and bends the RNA primer [17,56] to keep the same contacts with the sugar-phosphate backbone. It is probable that the requirement for similar binding of both types of duplexes explains a smaller footprint of Polα on the template:primer and a less extensive network of contacts, which results in a low affinity with a *K_d_* of ~320 nM for the RNA:DNA helix [20]. Its relatively weak interaction with the template:primer explains the high sensitivity of Polα to unconventional DNA structures, which is manifested by DNA synthesis abrogation on the certain templates [38,56,57]. It is likely that the limited Polα processivity on poly-dT templates is due to DNA bending and/or the triplex formation between the DNA:DNA duplex and the template’s 5′-tail [57,58], rather than to the intrinsic ability of Polα to count the amount of incorporated dNMPs [56]. Moreover, no Polα pausing was observed on DNA templates of random sequence [38,55].

## 6. Polα Inhibition by Aphidicolin

**Figure 8:**
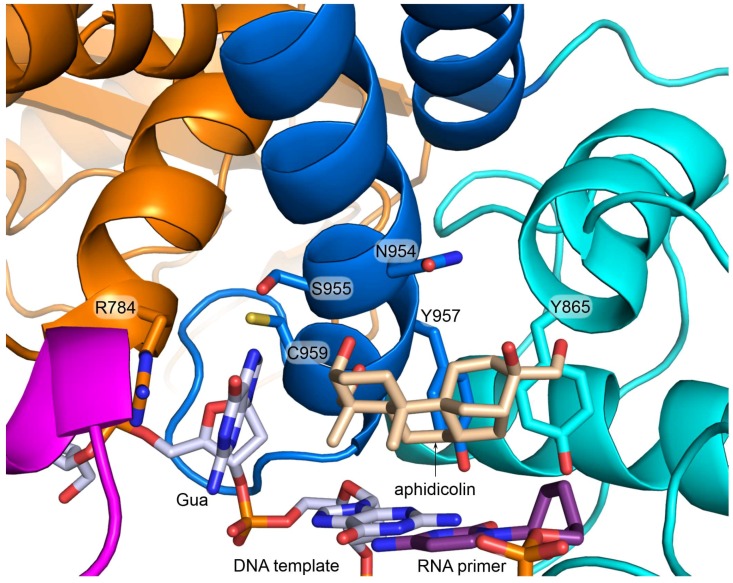
Close-up view of the Polα active site with bound aphidicolin and the DNA:RNA duplex. The color scheme for p180core subdomains is same as in Figure 7. The carbons of aphidicolin are colored wheat. Side chains of the key residues, participating in hydrophobic interactions with aphidicolin and in stabilization of the *syn* conformation of the templating guanine, are shown as sticks. RNA primer contains a dideoxy-cytidine at the 3′-end. This figure was drawn using the coordinates of the p180core/DNA:RNA/aphidicolin complex (PDB ID 4Q5V).

Aphidicolin, an antimitotic metabolite of the mold *Cephalosporium aphidicola*, is a potent inhibitor of DNA replication in a variety of organisms [59,60]. It specifically inhibits B-family DNA polymerases, with Polα being the most sensitive to it [61]. Aphidicolin demonstrated potent growth-inhibitory and cytotoxic activities against human tumor cell lines cultured in vitro, but the absence of structural information hampered the improvement of its inhibitory properties [62,63,64]. The structure of p180core in ternary complex with a DNA:RNA duplex and aphidicolin revealed the mechanism of Polα inhibition and provided the structural rationale for design of a new generation of drugs with superior solubility, stability, and inhibitory activity [17]. Aphidicolin binds Polα at the active site by occupying the hydrophobic pocket for a nascent base pair (Figure 8). The interaction between aphidicolin and Polα is mediated by an extensive pattern of hydrophobic contacts as well as by the hydrogen bonds between two oxygens and the main-chain nitrogens. Accommodation of the bulky “potato” shape of the inhibitor results in the fingers opening and *syn* conformation of the templating guanine due to the base rotation by 118° around the *N*-glycosidic bond. The preference of aphidicolin for purine at this position is due to stabilization of the *syn* conformation of a purine mediated by stacking with a side chain of Arg-784, by the hydrogen bond between N7 and Oγ of Ser-955, and by several van der Waals interactions. In contrast to the imidazole ring of a purine base, the larger pyrimidine ring would hardly fit the pocket formed mainly by a second α-helix of the fingers domain.

## 7. Mechanism of Concerted RNA-DNA Primer Synthesis by the Human Primosome

**Figure 9:**
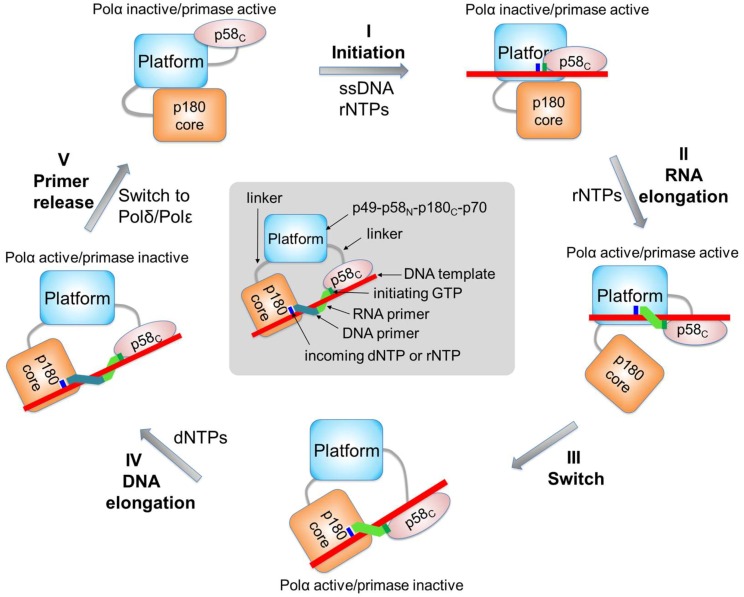
Schematic representation of conformational changes in the primosome during chimeric primer synthesis. At the first step (steps are labeled by roman numerals), p58_C_ moves toward p49 to initiate RNA synthesis. During the second step, p58_C_ moves toward p180core and pushes it to dissociate from the platform. Additionally, when RNA primer length is nine nucleotides, p58_C_ makes a steric hindrance with the platform, which prevents primer extension by p49. At the third step, p58_C_ rotates and loads the template:primer to the Polα active site. At the fourth step, Polα extends the RNA primer with dNTPs. At the fifth step, the primosome is replaced by Polε or Polδ.

The accumulated structural data allow for visualization of all key steps of the chimeric primer synthesis (Figure 9 and movie provided in [13]). The structure of the primosome in apo-form revealed the autoinhibited state of Polα due to p180core docking on the platform where the Zn2 module of p180_C_ and the OB domain of p70 are wedged into the template:primer-binding cleft of Polα [13]. During the initiation of RNA synthesis, p58_C_ binds the template and initiating rNTP and moves toward the active site of p49 residing on the platform. In the presence of the cognate, elongating rNTP at the catalytic site, the initiation complex is stabilized and proceeds toward the dinucleotide formation. While p58_C_ is important for primosome loading on early replication origins [65], it has low affinity for single-stranded DNA [43]. Presumably, other replication factors, like RPA, facilitate p58_C_ loading on the template [21]. During the RNA elongation step, p58_C_ moves toward p180core and pushes it to dissociate from the platform, resulting in Polα activation. The following primer extension results in a clash between p58_C_ and the platform that is responsible for RNA synthesis termination. At this step the interaction of p49 with a 9-bp DNA:RNA held by p58_C_ is compromised, leading to flotation of p58_C_/DNA:RNA and its capture by p180core floating nearby that results in the template-primer loading to the Polα active site. p58_C_ and p180core have an additional level of freedom relative to each other because they are independently connected with a platform by long linkers. According to modeling studies, these linkers allow Polα to generate a DNA track up to 20 nucleotides long, with p58_C_ holding the 5′-end of the primer. The weak grip of Polα on the DNA double helix could facilitate its displacement from the template:primer by RFC/PCNA or Polε.

Structural and biochemical data indicate that p58_C_ is a central mediator of all primosome transactions [13,42,43]. p58_C_ shuttles between the RNA- and DNA-polymerizing centers in the primosome, playing the role of the universal template:primer loader and regulator of primase and Polα. The linker between p58_N_ and p58_C_ allows p58_C_ to form the initiation complex with p49 during dinucleotide synthesis, to move away together with the 5′-end of the primer during its extension, and, finally, to intramolecularly transfer and load the template primed by a 9-mer RNA to the Polα active site. To perform these multiple duties, the p58_C_ shape conforms to several topological requirements: it is complementary to p49 during initiation and to p180core during the switch, and clashes with p58_N_ during RNA synthesis termination.

## 8. Concluding Remarks

The eukaryotic primosome was discovered more than 30 years ago [46,66,67,68] but its intricate mechanism of RNA-DNA primer synthesis has become clear only recently, owing to thrilling progress in structural studies. Comprehensive understanding of all primosome transactions, including initiation, elongation, and accurate termination of RNA synthesis, as well as primer transfer from primase to Polα, requires the crystal structures of the primosome in complex with a variety of substrates. Crystallization of these complexes is extremely challenging due to the size of the primosome and its significant flexibility. Fortunately, several key structures allowed for obtaining plausible three-dimensional models for all steps of chimeric primer synthesis. These structures include the human primosome in apo-form [13], the ternary complex p180core/DNA:RNA/dCTP [17], the binary complex p58_C_/DNA:RNA [13], complexes of p49–p58(19–253) or p49(1–390) with UTP [16,23], and full-length primase in apo-form [18]. Precise regulation of the concerted action of the two catalytic centers in the primosome is mainly based on the shape complementarity or the steric hindrance between its three components: a platform and two mobile domains, p58_C_ and p180core [13].

Further studies are required to understand the mechanism of primosome integration into the replisome and its regulation by other replicative factors. Studies in yeast have shown that trimeric Ctf4 links the N-terminal domain of the Polα catalytic subunit to the GINS complex, which is a part of the CMG helicase also containing Cdc45 and Mcm2–7 [25,69]. The helical N-terminal domain of p70 connected with the primosome by an 80-residue-long linker is a potential candidate for interaction with the replisome or regulatory proteins. It interacts with the hexameric helicase of SV40 large T antigen and activates the viral replisome [27,28]. Moreover, the N-terminal domain of the B subunit of Polε has a similar structure and interacts with the replisome [70,71]. A recent model of the replisome organization in *Saccharomyces cerevisiae* obtained from electron microscopy studies indicates that Polα is located behind the helicase, in proximity to both unwound parental strands [72]. High-resolution structural data are needed to build accurate replisome models (human-system models are more desirable) showing the primosome orientation and conformation during priming of the leading and lagging strands.

## Figures and Tables

**Table 1:** List of the high-resolution structures of the human primosome and its domains.

PDB ID	Resolution, (Å)	Protein Construct	Structural Metals	Cofactors	Deposition Date	Reference
3L9Q	1.7	p58(272–464)	4Fe-4S		5 January 2010	[21]
3Q36	2.5	p58(266–457)	4Fe-4S		21 December 2010	[22]
4BPU	2.7	p49 ^a^/p58(1–253)	Zn		28 May 2013	[16]
4BPW	3.0	p49 ^a^/p58(1–253)	Zn	UTP, Mg	28 May 2013	[16]
4BPX	3.4	p49 ^a^/p58(19–253) ^b^/p180(1445–1462)	Zn		28 May 2013	[16]
4LIK	1.7	p49(1–390) ^c^	Zn		2 July 2013	[23]
4LIL	2.6	p49(1–390) ^c^	Zn	UTP, Mn	2 July 2013	[23]
4MHQ	2.2	p49	Zn		30 August 2013	
4QCL	2.2	p180(335–1257) ^d^	Zn	DNA:RNA, dCTP, Mg	12 May 2014	[17]
4Q5V	2.52	p180(335–1257) ^d^		DNA:RNA, aphidicolin	17 April 2014	[17]
4RR2	2.65	p49/p58	Zn, 4Fe-4S		5 November 2014	[18]
4Y97	2.51	p180(1265–1444)/p70	Zn		17 February 2015	[19]
5DQO	2.3	p58(272–464) ^e^	4Fe-4S		15 September 2015	
5EXR	3.6	p49/p58/p180 ^d^/p70	Zn, 4Fe-4S		24 November 2015	[13]
5F0Q	2.2	p58(266–456)	4Fe-4S	DNA:RNA, Mg	28 November 2015	[13]
5F0S	3.0	p58(266–456)	4Fe-4S	DNA:RNA, Mn	28 November 2015	[13]
5IUD	3.3	p180(338–1255)		DNA:DNA	17 March 2016	[20]

^a^ Mutations Lys-72-Ala and Met-73-Ala; ^b^ N-terminus of p58 is fused to the primase-binding peptide of p180 via a 15 amino acid linker; ^c^ Residues 360–379 and 409–420 are deleted; ^d^ Mutation Val-516-Ala; ^e^ Mutation Tyr-347-Phe.
